# Supplementary material for: A systematic review of barriers and facilitators to antenatal screening for HIV, syphilis or hepatitis B in Asia: Perspectives of pregnant women, their relatives and health care providers
Source: PLoS One. 2024 May 31;19(5):e0300581. doi: 10.1371/journal.pone.0300581 (PMC11142523; doi:10.1371/journal.pone.0300581)
Supplement: S2 Table — (DOCX) [file pone.0300581.s002.docx]

| **Citation** | **Title Abstract** | **Background**  **Objectives** | **Qualitative approach** | **Researcher characteristics** | **Context** | **Sampling strategy** | **Ethical issues** | **Data collection methods/**  **instruments** | **Unit of study** | **Data processing** | **Data analysis** | **Trustworthiness technique** | **Synthesis** | **Links empirical data** | **Implications** | **Limitations** | **Conflicts of interest** | **Funding** | **Score (%)** |
| --- | --- | --- | --- | --- | --- | --- | --- | --- | --- | --- | --- | --- | --- | --- | --- | --- | --- | --- | --- |
| [21] | Yes | Yes | Yes | No | Yes | Yes | No | Yes | Yes | Yes | Yes | No | Yes | Yes | Yes | Yes | Yes | Yes | 85,71 |
| [41] | Yes | Yes | Yes | No | Yes | Yes | No | Yes | Yes | Yes | Yes | Yes | Yes | Yes | Yes | Yes | No | No | 80,95 |
| [26] | Yes | Yes | Yes | No | Yes | Yes | No | Yes | Yes | Yes | Yes | No | Yes | Yes | Yes | No | No | No | 71,43 |
| [22] | Yes | Yes | Yes | No | Yes | Yes | No | Yes | Yes | Yes | Yes | No | Yes | Yes | Yes | Yes | Yes | Yes | 78,26 |
