# Supplementary material for: A systematic review of barriers and facilitators to antenatal screening for HIV, syphilis or hepatitis B in Asia: Perspectives of pregnant women, their relatives and health care providers
Source: PLoS One. 2024 May 31;19(5):e0300581. doi: 10.1371/journal.pone.0300581 (PMC11142523; doi:10.1371/journal.pone.0300581)
Supplement: S3 Table — (DOCX) [file pone.0300581.s003.docx]

| **Citation** | **Title** | **Abstract** | **Background** | **Objectives** | **Study design** | **Setting** | **Participants** | **Variables** | **Data sources** | **Bias** | **Study size** | **Quantitative variables** | **Statistical methods** | **Participants** | **Descriptive data** | **Outcome data** | **Main results** | **Other analyses** | **Keys results** | **Limita-tions** | **Interpre-tation** | **Generali-sation** | **Funding** | **Score** |
| --- | --- | --- | --- | --- | --- | --- | --- | --- | --- | --- | --- | --- | --- | --- | --- | --- | --- | --- | --- | --- | --- | --- | --- | --- |
| [31] | Yes | No | Yes | Yes | Yes | Yes | Yes | No | No | No | No | No | No | No | Yes | Yes | Yes | No | Yes | No | Yes | No | Yes | 52,17 |
| [25] | Yes | Yes | Yes | Yes | Yes | Yes | Yes | Yes | Yes | Yes | Yes | Yes | Yes | No | Yes | Yes | Yes | Yes | Yes | Yes | Yes | Yes | Yes | 95,65 |
| [32] | Yes | No | Yes | Yes | Yes | No | Yes | No | No | No | Yes | No | No | Yes | Yes | Yes | Yes | No | Yes | No | No | No | Yes | 52,17 |
| [46] | Yes | Yes | Yes | Yes | Yes | Yes | Yes | Yes | Yes | No | Yes | No | No | Yes | Yes | Yes | Yes | Yes | Yes | Yes | Yes | Yes | Yes | 82,61 |
| [35] | No | No | Yes | No | Yes | Yes | Yes | No | No | No | Yes | No | No | No | Yes | Yes | Yes | No | No | Yes | Yes | Yes | Yes | 52,17 |
| [24] | Yes | Yes | Yes | Yes | Yes | Yes | Yes | Yes | Yes | No | Yes | No | Yes | No | Yes | Yes | Yes | No | Yes | Yes | Yes | Yes | Yes | 82,61 |
| [33] | Yes | No | Yes | Yes | Yes | Yes | Yes | Yes | Yes | No | Yes | No | Yes | Yes | Yes | Yes | Yes | No | Yes | Yes | Yes | Yes | No | 78,26 |
| [43] | Yes | No | Yes | Yes | Yes | Yes | Yes | No | No | Yes | No | No | Yes | No | Yes | Yes | Yes | No | Yes | Yes | No | Yes | No | 60,87 |
| [37] | No | No | Yes | Yes | Yes | Yes | Yes | No | No | No | Yes | No | No | Yes | Yes | Yes | Yes | No | Yes | No | Yes | Yes | Yes | 60,87 |
| [28] | No | No | Yes | No | No | No | No | No | No | No | No | No | No | No | Yes | Yes | Yes | No | No | No | Yes | No | Yes | 26,09 |
| [44] | Yes | Yes | Yes | Yes | Yes | Yes | Yes | Yes | Yes | Yes | Yes | Yes | Yes | No | Yes | Yes | Yes | Yes | Yes | Yes | Yes | Yes | Yes | 95,65 |
| [34] | Yes | No | Yes | Yes | Yes | Yes | Yes | No | No | No | Yes | Yes | No | No | Yes | Yes | Yes | No | Yes | Yes | Yes | Yes | Yes | 69,57 |
| [42] | Yes | Yes | Yes | Yes | Yes | Yes | Yes | No | Yes | No | Yes | No | Yes | Yes | Yes | Yes | Yes | Yes | Yes | Yes | Yes | Yes | No | 82,61 |
| [38] | Yes | Yes | Yes | Yes | Yes | Yes | Yes | No | No | No | No | No | Yes | No | Yes | Yes | Yes | No | Yes | Yes | Yes | Yes | Yes | 69,57 |
| [29] | Yes | No | Yes | Yes | No | No | No | No | No | No | No | No | No | No | Yes | No | Yes | No | Yes | No | Yes | No | Yes | 34,78 |
| [47] | Yes | Yes | Yes | Yes | Yes | Yes | Yes | Yes | Yes | No | Yes | Yes | Yes | Yes | Yes | Yes | Yes | Yes | Yes | Yes | Yes | Yes | Yes | 95,65 |
| [36] | Yes | Yes | Yes | Yes | Yes | Yes | Yes | No | No | No | No | No | No | No | Yes | Yes | Yes | No | Yes | Yes | Yes | Yes | No | 60,87 |
| [23] | Yes | Yes | Yes | Yes | Yes | Yes | Yes | Yes | Yes | No | No | Yes | Yes | No | Yes | Yes | Yes | No | Yes | Yes | Yes | No | Yes | 78,26 |
| [27] | Yes | Yes | Yes | No | Yes | Yes | Yes | Yes | No | No | Yes | Yes | Yes | No | Yes | Yes | Yes | No | Yes | Yes | Yes | Yes | Yes | 78,26 |
