# Supplementary material for: A systematic review of barriers and facilitators to antenatal screening for HIV, syphilis or hepatitis B in Asia: Perspectives of pregnant women, their relatives and health care providers
Source: PLoS One. 2024 May 31;19(5):e0300581. doi: 10.1371/journal.pone.0300581 (PMC11142523; doi:10.1371/journal.pone.0300581)
Supplement: S1 File — (DOCX) [file pone.0300581.s004.docx]

TITLE-ABS-KEY ( "barriers" OR "facilitators" OR "factors" AND ( "antenatal screening" OR "prenatal screening" OR "screening during pregnancy" OR "pre-natal screening" OR "antenatal testing" OR "prenatal testing" OR "testing during pregnancy" OR "pre-natal testing" OR ( "screening" AND "pregnant women" ) OR ( "testing" AND "pregnant women" ) OR ( "screening" AND "pregnant wives" ) OR ( "testing" AND "pregnant wives" ) ) AND ( "HIV" OR "syphilis" OR ( "HBV" OR "hepatitis B" ) ) AND ( "Asia" OR "Asian" OR "Afghanistan" OR "Armenia" OR "Azerbaijan" OR "Bahrain" OR "Bangladesh" OR "Bhutan" OR "Brunei" OR "Cambodia" OR "China" OR "Cyprus" OR "Georgia" OR "India" OR "Indonesia" OR "Iran" OR "Iraq" OR "Israel" OR "Japan" OR "Jordan" OR "Kazakhstan" OR "Kuwait" OR "Kyrgyzstan" OR "Laos" OR "Lebanon" OR "Malaysia" OR "Maldives" OR "Mongolia" OR "Myanmar" OR "Nepal" OR "North Korea" OR "Oman" OR "Pakistan" OR "Palestine" OR "Philippines" OR "Qatar" OR "Russia" OR "Saudi Arabia" OR "Singapore" OR "South Korea" OR "Sri Lanka" OR "Syria" OR "Taiwan" OR "Tajikistan" OR "Thailand" OR "Timor-Leste" OR "Turkey" OR "Turkmenistan" OR "United Arab Emirates" OR "Uzbekistan" OR "Vietnam" OR "Yemen" ) AND PUBYEAR AFT 2000)
