# Supplementary material for: A systematic review of barriers and facilitators to antenatal screening for HIV, syphilis or hepatitis B in Asia: Perspectives of pregnant women, their relatives and health care providers
Source: PLoS One. 2024 May 31;19(5):e0300581. doi: 10.1371/journal.pone.0300581 (PMC11142523; doi:10.1371/journal.pone.0300581)
Supplement: Attachment — Submitted filename: response_reviewers.docx [file pone.0300581.s005.docx]

Institute for Global Health

University College London

30 Guilford Street

London, WC1N 1EH

United Kingdom

Doctor Gifty Dufie Ampofo,

Academic Editor, *PLOS ONE*

Dear Doctor Ampofo,

We thank you and the reviewer for your time and generous comments provided on our manuscript PLONE-D-23-03027 entitled "Barriers and facilitators to antenatal screening for sexually transmitted diseases in Asia: a scoping review", and we thank you for the opportunity to address these comments. After consideration of your comments, we have made several improvements.

The comments provided are shown in bold below, with our responses in italics.

## Editor's Comments to the Author:

# 1. Please ensure that your manuscript meets PLOS ONE's style requirements, including those for file naming.

*Response: We have ensured that the manuscript meets the stylistic requirements of PLOS ONE.*

# 2. Please include a copy of Tables 4 and 5 which you refer to in your text on page 6.

*Response: A copy of tables 4 and 5 has been included under the name S2 Table and S3 Table.*

# 3. Please include captions for your Supporting Information files at the end of your manuscript, and update any in-text citations to match accordingly.

*Response: Captions for our Supporting Information files have been included at the end of the manuscript.*

## Reviewers’ Comments to the Author:

## Reviewer #1:

**The topic is of great interest, however the manuscript seems hurriedly put together. Concepts involved such as Andersen's model are not clearly and coherently applied in the course of the write-up.**

*Response: Thank you for your comment. We now define Andersen’s model (lines 148-153) and clearly and systematically apply the concepts cited. We have added Table 3 and systematically structured our results according to each component of Andersen’s model. The "facilitators" have been taken into account appropriately in the review.*

**The title for example does not capture the full essence of the review - it seems to be based on the "regional framework for the triple elimination of MTCT of HIV, Hep B and syphilis in Asia and Pacific" (lines 99 - 101)- that should have reflected clearly in the title. Such an understanding should have been provided briefly in the "Background" section of the script.**

**Secondly, the target population of the review was not clear right from the "title". Who did the review focus on - pregnant women, healthcare providers, relatives, etc. or all of them? That is not clear from the manuscript.**

*Response: We have changed the initial title from “Barriers and facilitators to antenatal screening for sexually transmitted diseases in Asia: a scoping review” to “A systematic review of barriers and facilitators to antenatal screening for HIV, syphilis or hepatitis B in Asia: perspectives of pregnant women, their relatives and health care providers.” The role of the WHO framework as the basis for this study has also been clarified in the background section (lines 76-89).*

**The objectives of the review as stated in lines 74 and 75 are not meant for a scoping review, but some other approaches that would generate clear evidence such as a review of qualitative evidence.**

*Response: On the basis of all the reviewers' comments, we decided to carry out a systematic analysis of the literature rather than a scoping review, using a second person to screen and review articles in duplicate and by searching other databases. We adapted the objectives to those of a systematic literature review (lines 113-115).*

**The methods section has lots of lapses.**

a. **A key concept such as the "population of the review" is not defined. The "search strategy" should have been systematically presented - e.g., keywords/ subject headings/ index terms that were used, an example of a search strategy of at least one of the databases should have been placed in the appendices; the search should have been as exhaustive as possible - searching two databases does not sound exhaustive enough.**

*Response: Thank you for this comment. To make the search as exhaustive as possible and in line with other reviews of this nature, we searched Ovid (MEDLINE, Embase, PsycINFO), Scopus, Global Index Medicus and Web of Science. Our database search strategy has been included in S1 File.*

b. **The "inclusion and exclusion criteria" should have been clearly defined each criterion - this subsection at best is confusing to the reader as it does not serve its purpose of clearly pointing out how studies were included in the review. Studies of experimental designs were excluded with no apparent reasons as to why they were.**

*Response: The eligibility criteria for the inclusion of studies have been rewritten and tabulated in Table 2 using the acronym SPlDER:* *S sample; P phenomenon of interest; D design; E evaluation; R research type. Our original inclusion/exclusion criteria were unchanged except that we also decided to include experimental design studies.*

c. **Under "Data extraction", the Andersen's framework was said to have been the guide - it would have been great to have this clearly "tabulated" with "findings" related to each component of the framework duly and systematically presented. The quality of studies was assessed, but this outcome did not feature further into any decision-making or discussion as to how study quality influenced the review process.**

*Response: We agree that a more systematic application of Andersen’s framework improves the paper so have rewritten the results section to clearly reflect each of the elements of the Andersen framework and added Table 3. As the aim of the review was to describe and synthesise a body of literature and not to determine effect size, we did not exclude studies on the basis of their quality assessment (lines 160-161) but we have summarised the quality assessment in S2 and S3 Tables to enable readers to see the quality of the evidence included in the review.*

d. **Andersen's model as defined in lines 123 - 124 does not seem to be in sync with the cited publication (11), i.e., predisposing, enabling, and needs factors. "Enabling factors" were not given the prominence required.**

*Response: We agree with this comment and have now given more explanation of the ‘enabling factors’ of antenatal screening (lines 151-152).*

**Aside the mention of "facilitators" on line 147, this key component of the review was hardly addressed. Furthermore in the results section, the nature of the findings as were presented did not necessarily aptly fit the defined factors under the Andersen model.**

*Response: As explained above we have now explicitly defined the "facilitators" in Table 1 and entirely rewritten the results section to follow the factors defined in Andersen’s model.*

**In its current form, this manuscript in my view is not fit for publication.**

*Response: Thank you for your detailed comments. We hope that the changes you have made will enable the study to be published.*

## Reviewer #2:

**Title…..perhaps the title should be restricted to HIV, Syphilis and Hepatitis B rather than ‘sexually transmitted diseases’.**

*Response: Thank you. We have changed the title to “A systematic review of barriers and facilitators to antenatal screening for HIV, syphilis or hepatitis B in Asia: perspectives of pregnant women, their relatives and health care providers.”*

**Introduction: Despite being a scoping review, a little more detail will improve the Background and situate arguments in better context.**

*Response: We have expanded the introduction to better place the study in context. We have also changed from a scoping to a systematic review.*

**Lines 50/51…….the authors can provide recent data on the morbidity and mortality they refer to…..first globally and in the Asian context Quantify the prevalence of these STDs in Asia (at least present data from some Asian countries).**

*Response: Data on mother-to-child transmission of HIV, syphilis and hepatitis B were added to the introduction. The prevalence of the STDs considered was quantified in the introduction section.*

**Lines 58/61…..can the authors assign, at least, an estimate of how many children are born to these STDs? Can they quantify antenatal screening for STDs in Asia? Can they give us an idea of how low is ‘low’.**

*Response: We have added an estimate of the number of children born with these STDs (lines 54-59), as well as a quantification of antenatal screening for STDs in Asia (lines 63-68).*

**I think it would be useful to give a brief overview of this WHO regional framework and what different prescriptions it gave compared to whatever existed before its formation**

*Response: Thank you for this suggestion. We have added an overview of the WHO regional framework and its main prescriptions (lines 76-87).*

**Lines 68/70……The authors may want to give more meaning to the listed ‘barriers’ and how they relate to uptake of HIV screening services. It would be particularly interesting to see what the story is for “health system and health care provider issues”. In many jurisdictions in sub-Saharan Africa, HIV screening is part of the antenatal care package and is offered using the opt-out model.**

*Response: Thank you for this suggestion. The obstacles listed have been detailed for greater clarity.*

**There is no literature review summarising the research-based evidence on barriers and facilitators to antenatal screening for STDs in the Asian context. Why is the statement above a problem? What is the burden of maternal and child morbidity and mortality in Asia in the context of HIV, Syphilis, Hepatitis C? What are the fall-outs from the supposed low uptake of available screening services? What do we stand to lose if this review is not done to better understand barriers and facilitators that can help inform useful interventions?**

*Response: We have clarified and expanded the introduction by highlighting the importance of this review (lines 88-92) and giving the reasons why a literature review in Asia is needed (lines 102-112). We now detail, in the introduction, the burden of maternal and infant morbidity and mortality in Asia within the limits of available data. We have also explained the consequences of the low uptake of screening services (lines 70-75).*

**Can the authors rewrite the Methods section without ‘We’?**

*Response: We decided to keep this section written in active voice as it is easier to understand and saves words. It is nowadays recommended for scientific writing in biomedical journals.*

**Line 84. We used a very inclusive search strategy to ensure that no item was missed…..Can you give a 100% guarantee no item was missed?**

*Response: We removed this sentence from the manuscript.*

**There appears to be something in Line 92 that is not supposed to be there.**

*Response: We removed this from the manuscript.*

**Inclusion and Exclusion Criteria: The inclusion and exclusion criteria appear to be ‘all over the place’. They can be made more focused. We did not include studies investigating antenatal screening for other STDs……..This does not qualify as an exclusion criterion because you have earlier specified that you are dealing with HIV, Hep B and Syphilis.**

*Response: We agree and have rewritten the eligibility criteria for study inclusion using the acronym SPlDER: S sample; P phenomenon of interest; D design; E evaluation; R research type.*

**The data extraction sub-section has nothing on “facilitators”**

*Response: In the methods, we have added a subsection on the extraction of data and explained how we focus upon both barriers and facilitators to antenatal screening within the structure of Andersen’s framework (Table 3).*

**Line 121…..the authors may want to justify the choice of Andersen’s conceptual model over other models they could have used.**

*Response: Thank you for your suggestion. We chose the Andersen conceptual model because it provides an understanding of how individuals and environmental factors influence health behaviours. This theoretical framework is widely used in literature reviews on healthcare utilisation. This has been justified in lines 147-150.*

**For Table 1, I think the year the study was conducted ought to be in separate column by itself. This will enable readers to relate them to implementation of the MDGs and contextualize them.**

*Responses: We agree with this suggestion and a separate column for the date has been added to Table 1.*

**I have some questions relating to Fig.1;**

**……you mentioned Google Scholar but I don’t see in the flow diagram**

**…….I am struggling to understand how you excluded 546 articles because the studies were not conducted in Asia when ‘Asia’ should have been a key part of your search strategy. Kindly elaborate on how this happened.**

*Responses: We have modified Figure 1 to reflect the new search strategy. With the new search strategy, only four articles were found to have been conducted outside Asia because the term "Asia" appeared in their abstracts.*

**Lines 210/211…….please give more meaning to ‘perceptions of poor healthcare support’ and ‘concerns about the reactions of healthcare workers**

*Response: We agree and have clarified the “perception of poor healthcare support” and the “concerns about the reactions of healthcare workers” (lines 276-291).*

**Secondly, of the 16 articles, 15 were on HIV and 1 was on Syphilis with nothing on Hepatitis B. In this context, I fail to see how you can make any reasonable pronouncements on Syphilis and Hepatitis B screening uptake. On this basis, I suggest you drop off Syphilis and Hep B and make your work entirely about the uptake of HIV screening than about STDs and appropriately reorient your discussion to that effect.**

*Response: We agree with your suggestion with respect to the discussion and conclusions and have rewritten these sections with respect to HIV only. However, the new search showed up three papers on syphilis and one on hepatitis B, so we believe it is important to highlight this gap and summarise the limited evidence in the results.*

We hope that you will be satisfied with the amendments made. If there are any further issues do not hesitate to get in touch. We would like to thank you again for your time and consideration of our manuscript.

Yours sincerely,

Lucie Sabin (on behalf of all co-authors)
