# Supplementary material for: A systematic review of barriers and facilitators to antenatal screening for HIV, syphilis or hepatitis B in Asia: Perspectives of pregnant women, their relatives and health care providers
Source: PLoS One. 2024 May 31;19(5):e0300581. doi: 10.1371/journal.pone.0300581 (PMC11142523; doi:10.1371/journal.pone.0300581)
Supplement: Attachment — Submitted filename: Review of PONE-D-23-03027_R1.docx [file pone.0300581.s006.docx]

**Manuscript Number:**

**Full Title:** A systematic review of barriers and facilitators to antenatal screening for HIV, syphilis or hepatitis B in Asia: perspectives of pregnant women, their relatives and health care providers.

**Authors:** Lucie Sabin Hassan Haghparast-Bidgoli Faith Miller Naomi Saville

**GENERAL COMMENTS:**

This review deals with an important topic that could be very useful in the prevention of HIV, syphilis and hepatitis B and transmission of these infections to infants. However, there are several major limitations to the quality of the review which hinder its relevance and applicability. The initial most striking feature of this review is that all four authors are affiliated with only one institute and this institute is in a high-income country that is not in Asia (used as general term here as the authors do not define Asia in their manuscript). Do any of these authors have lived experience of ANC, or healthy policy or practice in the region they are reviewing? If so, it would be helpful to have this information somewhere. Furthermore, the review only includes manuscripts published in English, a major limitation given the region being considered. there are excellent research Institutes throughout Asia, and no doubt this review would be enhanced if it included some collaborations within Asia to increase available grey literature and other studies that may not have been in their search methods.

**DETAILED COMMENTS:**

**ABSTRACT:**

“Despite improvements” is vague, some time reference of stats would be helpful here.

STIs is more commonly used now, rather than STDs.

“Antenatal screening” (Sabin, p. 1)

- and treatment. without treatment, screening won’t prevent transmission.

Methods paragraph. typo ‘conducted’ included twice in 1st and last sentence.

What is the definition of ‘Asia’? This should be included in the abstract.

Results section: please define in predisposing characteristics, enabling factors and need factors who you are referring to. The pregnant woman? The health worker?

**INTRODUCTION:**

“antenatal screening” (Sabin, p. 12) Line 62. And treatment, screening alone will not prevent transmission.

“infected women may transmit infections to their sexual partners or children” (Sabin, p. 13) Line 71. Please rephrase. Women are often infected by their partner, only saying women may give it to their partners overemphasizes their responsibility. What do you mean by infecting their children? Do you mean by MTCT? If so please be specific.

“Meanwhile, it encourages the participation of women living with HIV” (Sabin, p. 13) Line 86. prevention of MTCT of HIV, syphilis and hepatitis B is a shared responsibility, men and communities should also be encouraged to participate.

Preventing male transmission to women during sex, as well as preventing community transmission, of HIV, Syphilis and Hep B is also an effective method of preventing neonatal and infant infections. Whilst I recognise this is not the focus of the review, it should at least be mentioned to prevent misunderstandings and reduce stigma. Overly focusing on pregnant women being the source of transmission to their infants misses an opportunity to emphasise that they are not always the original source of the infection and may not have been able to negotiate appropriate protection for themselves in order to avoid infection.

“An estimated 10,000 new HIV infections occurred 56 among children aged 0–14 years in the Asia Pacific region in 2017” (Sabin, p. 12) Line 56/57. What is the number of infants infected with HIV due to MTCT? You mentioned 10,000 children infected between 0 and 14 years, but clearly not all of these are necessarily due to MTCT.

**METHODS:**

Line 122, word repetition.

**Research type.** Why were the articles limited to English? Given most countries in Asia have a primary language other than English this seems a big problem / barrier to identifying relevant research.

**RESULTS:**

**Table 3.** It would be helpful to also have a column of disease studied in this table.

Line 208. Whose knowledge are you referring to?

Paragraph re male partner’s opinion. In some countries mentioned it may be impossible for a woman to be screened without the express permission of the husband. It would be useful to contrast findings against legal framework for relevant countries as the approach to overcoming this barrier would be very different.

**DISCUSSION:**

Line 317. Given this review was limited to the English language I do not agree with it being referred to as a “comprehensive synthesis.”

Terminology used is not consistent regarding if this is a scoping review, narrative review or systematic review.

Paragraph 2. Part of the justification for this review was that findings in Asia may differ from that already published in sub-Saharan Africa. Given this, it would be interesting to understand the similarities and differences in more detail in this paragraph.

Line 330/331. Can you reference other differences in ANC screening or barriers that may support this statement?

Line 336/337. It is likely that training programs already exist, could you please highlight what efforts are already made in these settings before suggesting interventions. Again line 340/341 calls for free screening, this would be more helpful if information regarding whether this does or does not exist in the areas included in studies would be more meaningful.

The limitations paragraph needs to mention the limitation of including only English language and the apparent lack of inclusion of experts from the region.

**CONCLUSION:**

“and STDs” (Sabin, p. 30) Line 454. Please rephrase, you do not address all sexually transmitted infections.

“systematic review” (Sabin, p. 30) Line 354. Be consistent with use of terms narrative or systematic review.

In terms of translating these findings into practice it would be helpful, if possible, to comment in the conclusion as to which factors appeared to be the largest barriers. It may be that this varies in different countries, or at the sub district level. In addition to reviewing studies that look at implementing screening (and treatment), it would be more helpful to also know/contrast this with which countries have policies for ANC screening and treatment and if this is meant to be free or fee for service.
