# Supplementary material for: A systematic review of barriers and facilitators to antenatal screening for HIV, syphilis or hepatitis B in Asia: Perspectives of pregnant women, their relatives and health care providers
Source: PLoS One. 2024 May 31;19(5):e0300581. doi: 10.1371/journal.pone.0300581 (PMC11142523; doi:10.1371/journal.pone.0300581)
Supplement: Attachment — Submitted filename: response_reviewers.docx [file pone.0300581.s007.docx]

Institute for Global Health

University College London

30 Guilford Street

London, WC1N 1EH

United Kingdom

Dr Stephen Michael Graham,

Academic Editor, *PLOS ONE*

Dear Dr Graham,

We thank you and the reviewer for your time and generous comments provided on our manuscript PLONE-D-23-03027 entitled " A systematic review of barriers and facilitators to antenatal screening for HIV, syphilis or hepatitis B in Asia: perspectives of pregnant women, their relatives and health care providers", and we thank you for the opportunity to address these comments. After consideration of your comments, we have made several improvements.

The comments provided are shown in bold below, with our responses in italics.

## Reviewer #2:

**I am grateful to the authors for incorporating the previous comments made. The manuscript looks more refined now but will still need to be improved in some aspects as listed below. After they have worked on these comments, I believe the work can be accepted for publication.**

*Response: Thank you for your helpful comments. We have incorporated your suggestions to improve the manuscript for publication.*

**Abstract**

**• Line 21………I am wondering if Antenatal screening alone is enough for PMTCT. Shouldn’t it be accompanied by treatment?**

*Response: We have now acknowledged the importance of appropriate treatment as a complement to screening for effective prevention of mother-to-child transmission (line 21).*

**• Line 26…..read it again and rectify the grammatical error there….similar error in Line 30/31**.

*Response: We have now corrected the grammatical errors in line 27 and lines 31/32.*

**• Line 27……..what you sought for in those published articles should be in this line.**

*Response: We have now incorporated the relevant information sought from the published articles (lines 31/32).*

**• There is nothing about “barriers” in the Results section. If there is, it needs to be made more explicit.**

*Response: We have responded to this concern by explicitly mentioning barriers and facilitators in the revised version of the manuscript (line 39). No specification has been made in the abstract between barriers and enablers, as in most cases barriers can be the opposite of enablers (ie. being poor is a barrier and being rich an enabler).*

**Introduction**

**• Line 73/74……can the authors show proof that there is a low uptake of STDs screening?**

*Response: In the revised manuscript, we have included relevant information on screening coverage for the different diseases considered, providing tangible evidence to support the claim of low uptake (lines 72 to 82).*

**Methods**

**• Who or which persons conducted the initial search? (show with initials).**

*Response: LS conducted the initial search, and we specified it on page 7 line 148*

**• Who is the third reviewer?**

*Response: NS was the third reviewer, and we specified it in line 152.*

**• Line 133……what is the use of the boldened Error statement there?**

*Response: Thank you for drawing our attention to it. The reference problem has been resolved and the formatting corrected.*

**• In SPIDER, you defined SAMPLE to include women of childbearing age. I reckoned this work was about Antenatal Screening of Pregnant women. Please clarify the need to include women of childbearing age.**

*Response: Although our primary focus was antenatal screening in pregnant women, we chose to include women of childbearing age because many antenatal screening studies surveyed or interviewed not only pregnant women but also those who have already given birth or were of childbearing age and were recalling screening during pregnancy. We believe restricting the inclusion to studies that only sampled pregnant women could lead to the exclusion of studies relevant to our literature review.*

**• Review the Data Extraction section for some grammatical omissions and errors. Line 150 is missing “of”. Line 151 …..’categorizes’ instead of ‘categories’.**

*Response: We have reviewed the data extraction section and removed the grammatical omissions and errors (lines 156 and 159).*

**• Line 160……’that’ instead of ‘who’**

*Response: The mistake has been corrected now (line 169).*

**Discussion**

**• Line 321…..rephrase to read “…….allow conclusions to be drawn effectively about HIV alone”**

*Response: The sentence has been rephrased now (line 332).*

**• Line 340/341…….there is free screening of HIV, Hep B and Syphilis in many parts of sub-Saharan Africa already.**

*Response: Thank you for highlighting the importance of this contextual clarification. Free screening for HIV, hepatitis B and syphilis is also offered in many Asian countries, and this has been specified in lines 378 to 382.*

**Line 348……On heterogeneity……there were 19 quantitative studies and you could have evaluated heterogeneity statistically to enable you make a more refined pronouncement on the subject**

*Response: In this study, we aimed to identify factors affecting ANC screening reported in the literature in the region as we had expected, there were many heterogeneities in the design, variables included and analytical methods, which limited analysis and comparison of the results.*

**The Conclusion can be better written…..with emphasis on what specifically needs to be done and by which organization or department or health agency.**

*Response: We have now substantially revised the conclusion to emphasise our recommendations and the importance of health system stakeholder coordination at the national, regional, and local levels to implement effective interventions tailored to the different Asian contexts.*

## Reviewer #3:

**General comments**

**This review deals with an important topic that could be very useful in the prevention of HIV, syphilis and hepatitis B and transmission of these infections to infants. However, there are several major limitations to the quality of the review which hinder its relevance and applicability. The initial most striking feature of this review is that all four authors are affiliated with only one institute and this institute is in a high-income country that is not in Asia (used as general term here as the authors do not define Asia in their manuscript). Do any of these authors have lived experience of ANC, or healthy policy or practice in the region they are reviewing? If so, it would be helpful to have this information somewhere. Furthermore, the review only includes manuscripts published in English, a major limitation given the region being considered. there are excellent research Institutes throughout Asia, and no doubt this review would be enhanced if it included some collaborations within Asia to increase available grey literature and other studies that may not have been in their search methods.**

*Response: Thank you for raising these concerns. While the four authors are all affiliated to UCL, Naomi Saville has lived and worked for over 25 years in Nepal and is married into a Nepalese family with many relatives and friends that have direct experience of ANC as recipients and providers of services. Her research since 2005 has been mainly focused on improving maternal and child health in the South Asia region. Hassan Haghparast-Bidgoli has worked for over 10 years on public health trials in the region, in particular, Bangladesh and India.*

*Regarding the issue of language limitations, given the diversity of languages in Asia and due to resource constraints, we have decided to include only published studies in English. Importantly, in South and Southeast Asia local scientific journals are in English, which is our main geographical area of interest. However, we have acknowledged this in the limitations (lines 390/391).*

**Abstract**

**“Despite improvements” is vague, some time reference of stats would be helpful here.**

*Response: Due to the word limit for the abstract, it has not been possible to give detailed statistics. However, specific figures have been provided in the introduction section for a better understanding of the context (lines 53 to 57).*

**STIs is more commonly used now, rather than STDs.**

*Response: We have now replaced " STDs" with " STIs" throughout the manuscript, as recommended.*

**“Antenatal screening” (Sabin, p. 1) and treatment. without treatment, screening won’t prevent transmission.**

*Response: In the revised manuscript, we have now recognised the importance of treatment to complement antenatal screening and prevent transmission (lines 66, 69 and 369).*

**Methods paragraph. typo ‘conducted’ included twice in 1st and last sentence.**

*Response: We have now corrected the typo.*

**What is the definition of ‘Asia’? This should be included in the abstract.**

*Response: We have used the classification and definition of regions by the United Nations in our review to determine which countries to include. We have added more details on this in the methods section (Table 1) as the word limit does not allow us to add more details in the abstract.*

**Results section: please define in predisposing characteristics, enabling factors and need factors who you are referring to. The pregnant woman? The health worker?**

*Response: The predisposing characteristics and need factors refer explicitly to pregnant women. The enabling factors are differentiated, some concerning healthcare professionals and other pregnant women, as specified in brackets. We have tried to make this clearer in the abstract lines 39 and 41.*

**Introduction**

**“antenatal screening” (Sabin, p. 12) Line 62. And treatment, screening alone will not prevent transmission.**

*Response: In the revised manuscript, we now recognise the importance of treatment to complement antenatal screening and prevent transmission (lines 66, 69 and 369).*

**“infected women may transmit infections to their sexual partners or children” (Sabin, p. 13) Line 71. Please rephrase. Women are often infected by their partner, only saying women may give it to their partners overemphasizes their responsibility. What do you mean by infecting their children? Do you mean by MTCT? If so please be specific.**

*Response: Thank you for your comment. We have made the necessary changes to the manuscript to address these concerns by recognising that both men and women can be infected and by emphasising the mode of transmission without attributing responsibility solely to women (lines 82/83). In addition, we have clarified that the reference to children relates specifically to mother-to-child transmission (MTCT).*

**“Meanwhile, it encourages the participation of women living with HIV” (Sabin, p. 13) Line 86. prevention of MTCT of HIV, syphilis and hepatitis B is a shared responsibility, men and communities should also be encouraged to participate.**

*Response: In response to your helpful suggestion, we have made changes (lines 98/99) to explicitly specify that participation and responsibility extend beyond women to include men and communities. However, the WHO framework focuses especially on women and does not address the involvement of men living with HIV, syphilis and hepatitis B.*

**Preventing male transmission to women during sex, as well as preventing community transmission, of HIV, Syphilis and Hep B is also an effective method of preventing neonatal and infant infections. Whilst I recognise this is not the focus of the review, it should at least be mentioned to prevent misunderstandings and reduce stigma. Overly focusing on pregnant women being the source of transmission to their infants misses an opportunity to emphasise that they are not always the original source of the infection and may not have been able to negotiate appropriate protection for themselves in order to avoid infection.**

*Response: Thank you for highlighting the importance of recognising male transmission and community-based prevention of HIV, syphilis and hepatitis B in the prevention of neonatal and childhood infections. In response, we have revised the manuscript to include a brief mention of these aspects (lines 66/67 and 399).*

**“An estimated 10,000 new HIV infections occurred 56 among children aged 0–14 years in the Asia Pacific region in 2017” (Sabin, p. 12) Line 56/57. What is the number of infants infected with HIV due to MTCT? You mentioned 10,000 children infected between 0 and 14 years, but clearly not all of these are necessarily due to MTCT.**

*Response: We have responded to this concern by amending the manuscript and replacing the figure to provide a more accurate representation of the MTCT scenario in the region (lines 58 to 61).*

**Methods**

**Line 122, word repetition.**

*Response: We modified the sentence to avoid repetition.*

**Research type. Why were the articles limited to English? Given most countries in Asia have a primary language other than English this seems a big problem / barrier to identifying relevant research.**

*Response: We only reviewed studies and grey literature published in English due to resource constraints. We have now acknowledged this in the limitations (lines 390/391).*

**Results**

**Table 3.** **It would be helpful to also have a column of disease studied in this table.**

*Response: We have taken your comments into account by adding a column specifying the diseases studied in Table 3.*

**Line 208. Whose knowledge are you referring to?**

*Response: We have specified that the knowledge referred to is that of pregnant women (lines 217-8).*

**Paragraph re male partner’s opinion. In some countries mentioned it may be impossible for a woman to be screened without the express permission of the husband. It would be useful to contrast findings against legal framework for relevant countries as the approach to overcoming this barrier would be very different.**

*Response: To keep the results section focused on the results of the literature review, we added details to the discussion section on husbands' opinions (lines 374 to 376). We have not found any policies or legal frameworks that explicitly require the husband's authorisation to undergo screening, but in practice, due to social pressure or cultural norms, permission from the husband may be required.*

**Discussion**

**Line 317. Given this review was limited to the English language I do not agree with it being referred to as a “comprehensive synthesis.”**

*Response: We have replaced “comprehensive synthesis” with “narrative synthesis to keep the terminology consistent throughout the manuscript.*

**Terminology used is not consistent regarding if this is a scoping review, narrative review or systematic review.**

*Response: We are sorry if our terminology was unclear. We conducted a systematic review, characterised by a structured and rigorous methodology but the results are presented using a narrative synthesis. We have now made sure that the terminology is consistent throughout the manuscript, referring to a systematic review with narrative synthesis (not a narrative review or a scoping review).*

**Paragraph 2. Part of the justification for this review was that findings in Asia may differ from that already published in sub-Saharan Africa. Given this, it would be interesting to understand the similarities and differences in more detail in this paragraph.**

*Response: We have expanded the second paragraph of the discussion by highlighting the similarities and differences between our results and those obtained in the context of sub-Saharan Africa (lines 333 to 353).*

**Line 330/331. Can you reference other differences** **in ANC screening or barriers that may support this statement?**

*Response: We have expanded the paragraph starting on line 354 to highlight the differences in the context of ANC screening in different Asian countries (lines 354 to 364).*

**Line 336/337. It is likely that training programs already exist, could you please highlight what efforts are already made in these settings before suggesting interventions. Again line 340/341 calls for free screening, this would be more helpful if information regarding whether this does or does not exist in the areas included in studies would be more meaningful.**

*Response: The entire paragraph (lines 365 to 387) has been rewritten to highlight the interventions already carried out in Asia and the successes achieved in the region in terms of antenatal screening for sexually transmitted infections.*

**The limitations paragraph needs to mention the limitation of including only English language and the apparent lack of inclusion of experts from the region.**

*Response: The limitation of only including work published in English has now been acknowledged in the limitations paragraph (lines 390/391).*

**Conclusion:**

**“and STDs” (Sabin, p. 30) Line 454. Please rephrase, you do not address all sexually transmitted infections.**

*Response: STDs here refer to the STG objectives concerning HIV and STDs (lines 102 to 104).*

**“systematic review” (Sabin, p. 30) Line 354. Be consistent with use of terms narrative or systematic review.**

*Response: We have now tried to make it clear that we conducted a systematic review with results are presented using a narrative synthesis.*

**In terms of translating these findings into practice it would be helpful, if possible, to comment in the conclusion as to which factors appeared to be the largest barriers. It may be that this varies in different countries, or at the sub district level. In addition to reviewing studies that look at implementing screening (and treatment), it would be more helpful to also know/contrast this with which countries have policies for ANC screening and treatment and if this is meant to be free or fee for service.**

*Response: We have rewritten the conclusion to highlight the factors that appeared to be the largest barriers (lines 397/398) and our recommendations (lines 398 to 409). We have also revised the discussion session to include a discussion of countries' policies on antenatal screening and treatment (lines 377 to 382).*

We hope that you will be satisfied with the amendments made. If there are any further issues do not hesitate to get in touch. We would like to thank you again for your time and consideration of our manuscript.

Yours sincerely,

Lucie Sabin (on behalf of all co-authors)
