# Supplementary material for: A systematic review of barriers and facilitators to antenatal screening for HIV, syphilis or hepatitis B in Asia: Perspectives of pregnant women, their relatives and health care providers
Source: PLoS One. 2024 May 31;19(5):e0300581. doi: 10.1371/journal.pone.0300581 (PMC11142523; doi:10.1371/journal.pone.0300581)
Supplement: Attachment — Submitted filename: response_reviewers.docx [file pone.0300581.s008.docx]

Institute for Global Health

University College London

30 Guilford Street

London, WC1N 1EH

United Kingdom

Dr Stephen Michael Graham,

Academic Editor, *PLOS ONE*

Dear Dr Graham,

We thank you and the reviewer for your time and comments provided on our manuscript PLONE-D-23-03027 entitled " A systematic review of barriers and facilitators to antenatal screening for HIV, syphilis or hepatitis B in Asia: perspectives of pregnant women, their relatives and health care providers", and we thank you for the opportunity to address these comments. After consideration of your comments, we have made several improvements.

The comments provided are shown in bold below, with our responses in italics.

**Journal Requirements:**

**Please review your reference list to ensure that it is complete and correct. If you have cited papers that have been retracted, please include the rationale for doing so in the manuscript text, or remove these references and replace them with relevant current references. Any changes to the reference list should be mentioned in the rebuttal letter that accompanies your revised manuscript. If you need to cite a retracted article, indicate the article’s retracted status in the References list and also include a citation and full reference for the retraction notice.**

*Response: The list of references has been examined. It is complete and correct, and no changes were required.*

**Additional Editor Comments:**

**Thanks for resubmitting and addressing the comments of the reviewers so comprehensively.**

**I support publication - however, would it be possible to improve the reporting of these populations by age groups that they represent? Is that something that could be added in a specific column in Table for each study: age range OR what proportion were adolescent pregnancy for example.**

**Adolescent pregnancy is still common in the region and a very vulnerable population with known poorer outcomes for mother and baby. V neglected population and as at risk for such infections as other pregnant women but perhaps even less likely to be screened? There is a data gap.**

**If this is not possible, it may still be worth a comment in discussion to highlight lack of data by age, especially in this vulnerable group. a suggested ref for this would be Sabet F, et al. The forgotten girls: .....Lancet. 2023;402:1580-1596.**

*Response: Thank you for highlighting the importance of this research gap. Unfortunately, it was not possible to report the proportion of adolescent pregnancies in each study, as this information was not always included in the articles. However, we have added a paragraph in the discussion section on the lack of age-specific data, particularly for this vulnerable group of pregnant women, and the importance of filling this data gap (lines 388 to 394).*

We hope that you will be satisfied with the amendments made. If there are any further issues do not hesitate to get in touch. We would like to thank you again for your time and consideration of our manuscript.

Yours sincerely,

Lucie Sabin (on behalf of all co-authors)
